# The Effects of Olive Cake and Linseed Dietary Supplementation on the Performance, Carcass Traits, and Oxidative Stability of Beef from Young Podolian Bulls

**DOI:** 10.3390/ani15152188

**Published:** 2025-07-25

**Authors:** Paolo De Caria, Luigi Chies, Giulia Francesca Cifuni, Manuel Scerra, Francesco Foti, Caterina Cilione, Paolo Fortugno, Miriam Arianna Boninsegna, Corinne Giacondino, Salvatore Claps, Pasquale Caparra

**Affiliations:** 1 Department of Agriculture, Mediterranean University of Reggio Calabria, Via dell’Università, 25, 89124 Reggio Calabria, Italy; paolo.decaria@unirc.it (P.D.C.); lchies@unirc.it (L.C.); manuel.scerra@unirc.it (M.S.); francesco.foti@unirc.it (F.F.); caterina.cilione@unirc.it (C.C.); frtpla99s23h224h@studenti.unirc.it (P.F.); miriam.boninsegna@unirc.it (M.A.B.); corinne.giacondino@unirc.it (C.G.); 2 Council for Agricultural Research and Economics-Research Centre for Animal Production and Aquaculture, S.S.7 Via Appia, 85051 Bella Muro, Italy; giuliafrancesca.cifuni@crea.gov.it (G.F.C.); salvatore.claps@crea.gov.it (S.C.)

**Keywords:** shelf life, bull meat, olive pomace, flaxseed meal, local breeds, growth performance, slaughter performance, olive polyphenol

## Abstract

To evaluate animal performance and meat quality, stoned olive cake and linseed were used in an experimental test conducted on thirty-six young Podolian bulls, divided into four groups: the control group (CON), OC group (with olive cake containing a 30% as-fed basis of stoned olive cake), EL group (with linseed containing a 15% as-fed basis of extruded linseed), and OCEL group (with olive cake + linseed containing 20% stoned olive cake and 10% extruded linseed). The results show that olive cake supplementation did not influence performance in vita or the post-slaughter animal measurements (final body weight, DMI, FCR, ADG, carcass weight, dressing percentage, and pH) (*p* > 0.05); this was not true of the TBARS and color measurements, for which the meat samples showed excellent values (*p* < 0.001), especially in diets supplemented with olive cake. In conclusion, incorporating olive cake and linseed into the diet of fattening cattle may be a way to utilize a by-product of the olive industry and naturally increase the nutritional value of meat and meat-based products in Mediterranean regions. This would reduce environmental impacts and promote the valorization of this local feed source in alignment with the principles of the circular economy.

## Simple Summary

This study investigated the effects of dietary supplementation with stoned olive cake and extruded linseed on growth performance and meat quality in thirty-six Podolian bulls. Animals were randomly allocated to four dietary groups: a control (CON), an olive cake group (OC, 30% as-fed), a linseed group (EL, 15% as-fed), and a combined olive cake–linseed group (OCEL, 20% olive cake + 10% linseed as-fed). Productive parameters, including final body weight, dry matter intake, feed conversion ratio, average daily gain, carcass weight, and dressing percentage were unaffected by dietary treatments (*p* > 0.05). In contrast, meat from bulls receiving olive cake—alone or in combination—exhibited significantly improved oxidative stability and colorimetric parameters, as indicated by lower TBARS values and enhanced color (*p* < 0.001). These findings suggest that the inclusion of olive cake and linseed in the finishing diets of Podolian cattle may improve meat quality attributes without compromising animal performance. Moreover, the valorization of olive industry by-products through animal nutrition supports sustainability goals and the principles of the circular economy, particularly within Mediterranean production systems.

## 1. Introduction

It is paramount that alternate solutions are discovered for the disposal of by-products derived from the agri-food sector, given the increasing global population and the consequent need for a prudent food and water resource use.

With ambitious plans being implemented for a circular economy plan in recent years, international politics has begun shifting toward a more sustainable economy with the goal of minimizing waste and reusing by-products. Reintroducing waste into the production cycle is the main goal, as this allows for fresh resources to be obtained that can be utilized to create new goods and provide economic value [1].

Additionally, waste management is a crucial component of plans to lower greenhouse gas emissions, air and water pollution, and health issues.

Global warming is regarded as having a number of primary causes, including the cattle industry.

The main global sources of GHG emissions in the production of feed are land use, transport, and cultivation and processing.

In this case, incorporating agro-industrial waste products into animal feed might be a key element of a global effort to lessen the environmental effects of both livestock production and agro-industrial activities.

Some of these by-products are abundant in bioactive chemicals and may serve as an excellent supply of fresh goods, with applications in the circular economy and sustainability efforts [2]. The objective of a Zero Waste Economy is to reincorporate waste into the production cycle to be used as a new resource [3].

By-products from the agri-food business may be reused by turning them into zootechnical feed, which has economic benefits and a positive socio-environmental effect [4].

Since some by-products contain bioactive compounds, such as vitamins, minerals, phenolic compounds, and unsaturated fatty acids, using agro-industrial by-products in livestock can improve product quality and shelf stability, in addition to lowering the carbon footprint of animal products and the environmental problems caused by by-product accumulation [4,5,6].

There are key areas of the feed sector in which technological innovation is advantageously used for the reuse of by-products and agri-food residues, such as the enhancement of nutritional and functional qualities in food. Certain agro-industrial by-products are rich in bioactive compounds with demonstrated functional properties, including antioxidant, immunomodulatory, and metabolic effects. Beyond their nutritional contribution, these materials can be strategically incorporated into animal diets to improve both animal health and the quality of derived products. The inclusion of agro-industrial by-products in animal diets has been reported to improve the fatty acid profile, oxidative stability, and shelf life of meat, without affecting animal performance [7,8]. The livestock sector may benefit economically and ecologically from the use of agro-industrial by-products in animal feed, which will increase its sustainability and profitability. One of the most significant agro-industrial by-products in the Mediterranean region, and a significant pollutant from the manufacturing of olive oil, is olive cake (OC). In particular, recent studies have explored the recovery of bioactive phenolic compounds from olive oil by-products and their application in animal feeding strategies, with promising results in terms of sustainability and functional benefits [9]. Interest in OC research has grown as more has been revealed about the environmental issues associated with OC management and the commercial value of components such as phenols [10].

One of the potential final applications of this research is in animal feed, which would support the circular economy of Mediterranean nations.

The type of olive, the quantity of its components (skin, pulp, and stone), and the method used to extract the oil all have an impact on the chemical characteristics of olive cake [11]. Olive cake, beyond being a source of bioactive polyphenolic compounds, represents a high-energy feed ingredient owing to its notable content of residual oil, particularly rich in oleic acid. Pigs [12,13,14], cattle [15], small ruminants [16,17], broilers [18], and rabbits [19] have all been demonstrated to have positive responses to its use. The addition of olive cake to the diet of pigs may promote better growth, decrease the thickness of carcass fat, or give ruminants more energy [20,21].

Therefore, in order to reduce feeding costs while concurrently introducing a dietary source of oleic acid and bioactive compounds with antioxidant properties that may contribute to improved meat quality, stoned olive cake could be used as a partial substitute for conventional feed ingredients such as cereal grains.

Based on these considerations, linseed (*Linum usitatissimum*) was included in the present study alongside stoned olive cake, with the aim of further enhancing the nutritional and functional properties of the diet. Animal feed linseed has a high oil content (40%), with an α-linolenic acid content of approximately 55% with a 55% α-linolenic acid (ALA, 18:3n−3) ratio [22]. When linseed is added to ruminant diets, the amount of polyunsaturated fatty acids in animal products increases, particularly that of omega-3 PUFAs [23]. A range of FAs can be found in ruminant products. Some of them, such as omega-3 polyunsaturated fatty acids, may have positive effects on human health. Both 18:3 n-3 and omega-3 fatty acids with 20 and 22 carbons make up the omega-3 fatty acids that are found in beef. According to Mills et al. [24], omega-3 fatty acids, especially those with 20 and 22 carbons, may lower the risk of cardiovascular disease.

Today, consumers are more interested in natural preservatives than ever before, given their desire for products that are good for their health. Hence, supplying meals with a high nutritional value entails not only substituting natural antioxidants for synthetic preservatives, but also enhancing the health benefits and shelf life of the product.

The present study aimed to assess the effect of stoned olive cake supplementation, alone or in combination with extruded linseed, on the growth performance, carcass traits, and oxidative stability of beef from young Podolian bulls.

## 2. Materials and Methods

### 2.1. Ethical Statement

The experimental procedures in this experiment were evaluated and approved by the Animal Welfare Committee of the Mediterranean University of Reggio Calabria (Protocol Number 3015/2023) and carried out according to international guidelines on the protection of animals used for scientific research (Directive 2010/63/EU).

### 2.2. Animal Management, Dietary Treatments, and Sample Collection

The experiment was conducted from February to July 2023 on a family farm (project leader) located in Melissa (province of Crotone, Calabria region, Southern Italy) at 80 m above sea level (latitude: 39°17′46.712″ N, longitude: 17°1′34.651″ W), which breeds Podolian cattle for meat production.

Podolian is an autochthonous cattle breed from southern Italy; it is especially found in the regions of Calabria and Basilicata, and is particularly rustic, long-lived, and well-adapted to live in difficult environments with poor-quality vegetation (native pastures and shrubs). It is farmed with extensive or semi-extensive systems, still using the traditional practice of transhumance, which consists of the seasonal migration of livestock who move along the ancient pathways from the mountainous areas to those of the plains during the winter season, or conversely during the summer season, in order to find more suitable pastures.

Podolian cattle breeding is characterized by a cow–calf line in which calves are raised on natural pasture with their mothers and naturally weaned when they are about 7 to 8 months old. After weaning, they continue to be kept on pasture until 10–11 months of age and are then moved to housing systems made up of open barns with pens to be finished with concentrates and straw until slaughter at 15–16 or 17–18 months of age.

The experiment involved 36 young Podolian bulls that were randomly allotted to four equal groups of 9 animals each, in accordance with dietary treatments and balanced to their age (average initial age of 315.73 ± 19.40 days; mean ± SD) and body weight (average initial body weight of 304.31 ± 13.50 kg; mean ± SD).

The Podolian calves for each treatment were housed within the same newly constructed free-stall barn in separate contiguous pens with a feeding/resting area and an uncovered exercise area with a total space allowance of 14.5 m^2^ per head. Each pen was equipped with individual cribs and mangers with approximately 0.80 m feeding space per animal.

The animals in each group were fed the same basal forage consisting of wheat straw offered ad libitum once daily at 7.30 a.m., and one of the following pelleted experimental concentrates: (i) a concentrate without olive cake and extruded linseed supplementation (concentrate CTRL); (ii) a concentrate containing a 30% as-fed basis of stoned olive cake without extruded linseed supplementation (concentrate OC); (iii) a concentrate containing a 15% as-fed basis of extruded linseed without stoned olive cake supplementation (concentrate EL); and (iv) a concentrate containing 20% stoned olive cake and 10% extruded linseed (as-fed basis) supplementation (concentrate OCEL) (Table 1).

The experimental concentrates were given to each animal twice a day at 8.00 a.m. and 4:00 p.m., at a rate of 1.7 kg (as-fed basis) per 100 kg of live weight, and were consumed in their entirety by all of the animals. The concentrate offering was adjusted every two weeks according to the most recent body weight measurement. The amounts of wheat straw offered to and refused by each bull were weighed and recorded weekly. Water was given ad libitum for all treatments. The concentrates used in the experiment were formulated to be isonitrogenous and isoenergetic. The fattening period lasted for 170 days, including 20 days for the adaptation period and 150 days for the experimental period. Throughout the entire experimental period, the general health conditions of the animals remained optimal, and there was no onset of alterations of any kind, mainly with regard to the digestive system. The olive cake used in the experiment was provided by an olive farm with organic certification (project partner) located in Belcastro (province of Catanzaro, Calabria, southern Italy), which grows and processes olives into organic higher-quality extra virgin oil, and consequently also produces organic olive cake. In our study, we chose to use organic olive cake, free of synthetic chemical residues, so as not to compromise the extremely positive image of the extensive Podolian farming system, which uses environmentally and animal-friendly farming methods that supply high-quality products, and which is greatly appreciated by consumers.

The olive cake was obtained from a continuous two-and-a-half-phase olive oil mill plant (Pieralisi Group S.p.A., Jesi, Italy) by means of “cold extraction” (during the oil-making process, the temperature never exceeds 27 °C) according to the following operative conditions: olive crushing was carried out with a hammer crusher equipped with an inverter for the adjustment of its rotating speed, which, due to its particular construction characteristics, is able to reduce the thermal and oxidative stress on the product; olive paste malaxation (kneading) lasted about 45 min at 24–26 °C; and oil extraction was performed using a centrifugal extractor (two-and-a-half-phase decanter) Scorpion 5.9 model (Pieralisi Group S.p.A., Jesi, Italy), which allows for significantly reducing water consumption compared with conventional processes, while ensuring a higher concentration of polyphenols in both the oil and the olive cake. With this technology, the moisture content of the olive cake obtained was roughly 50%.

In order to avoid high losses of phytochemicals, antioxidants, and other bio-active compounds, in this experiment, we chose to dry the wet olive cake at room temperature using a green, simple, low-cost method developed by a small local company (project partner) located in Belcastro (province of Catanzaro, Calabria, southern Italy), with our support. In detail, after olive oil extraction, the fresh virgin olive cake was quickly transported from the oil mill to the naturally ventilated greenhouses, covered with UV-stabilized polyethylene, to dry. In these polyhouses, the olive cake was spread on the concrete floor in layers of about 10–12 cm and periodically mixed mechanically to speed up the drying process. The drying took place through natural air convection without using any additional systems. The drying process took 8–10 days, depending on the initial humidity of the olive cake. Once drying was completed, the olive cake was stoned, using a vibration sieve with a mesh size of 2 mm, and immediately pelleted and packaged by the local industry partner to ensure the highest quality, and it was subsequently sent to a feed mill for the production of pelleted experimental concentrates (Table 1).

After drying and pitting, several representative samples of the olive cake were collected and brought to the laboratory to detect the presence of mycotoxins and other potential contaminants.

The total amounts of feed consumed (concentrate and straw) by each animal were recorded and analyzed for dry matter (DM) content (in a forced air heater at 105 °C) to assess the dry matter intake (DMI). During the experimental period, the bulls were individually weighed every two weeks with a cattle scale to adjust the concentrate offering and to determine the average daily gain (ADG) and feed conversion ratio (FCR) values.

Representative samples of the concentrates, wheat straw, and used dried stoned olive cake were collected during the course of the experiment to be submitted to the laboratory for analysis.

### 2.3. Slaughter Procedures, Carcass Characteristics, and Meat Sampling

All bulls were humanely slaughtered on the same day, in a commercial slaughterhouse after a 60 km truck journey, after one night of fasting, but with access to water. All animals were slaughtered by means of throat cutting after captive bolt stunning. The slaughter procedures used comply with welfare regulations and the EU Council Regulation (EC) No 1099/2009 (EC, 2009) [25].

After slaughtering, the carcasses were weighed to obtain hot carcass weight and hot dressing (calculated as the relation between hot carcass weight and slaughter body weight). The carcasses were then halved and subjectively assessed for conformation and fatness in accordance with the European Union beef carcass classification system under Council Regulation EU No. 1308/2013 [26]. Each score was then further classified as high (+), medium (=), and low (−). Both the conformation and fatness scales ranged from 18 (S+, very good) to 1 (P−, very poor) and 15 (5+, very high) to 1 (1−, extremely low), respectively. Once the surveys were completed, the carcasses were stored in a refrigeration room at 2 ± 2 °C, following the routine refrigeration procedure used in the slaughterhouse. After refrigeration for 24 h, the carcasses were weighed again and the cold dressing percentage and cooling loss were calculated. Subsequently, around the 9th rib of the right carcass, the subcutaneous fat color was measured in triplicate as the CIELAB coordinates L*, a*, and b* according to the CIE system, using a portable colorimeter (Minolta CR300, Model CR-300, Minolta Co., Ltd., Osaka, Japan), illuminant D65, calibrated to a standard white tile.

Carcass pH was measured at 1 and 24 h postmortem using a portable pH-meter (HI 99163, Hanna Instruments, Woonsocket, RI, USA) equipped with a penetrating electrode and inserted approximately three cm into longissimus thoracis muscle between the 7th and 8th thoracic vertebrae. The electrode was calibrated to 14 °C (abattoir temperature) with two buffers (pH 7.0 and pH 4.0).

After pH testing following 48 h of refrigeration, the part of the LD (Longissimus Dorsi), in a cranial–caudal direction, between the 6th and 10th thoracic vertebra, was taken from each right carcass for the analysis of lipid oxidation (TBARSs) and color.

### 2.4. Feed and Analysis Meat Proximate Analysis

Crude protein, crude fat, and crude ash were measured in feed samples using 1995 AOAC procedures 984.13, 920.39, and 942.05, respectively [27]. Analysis of the fiber fractions (NDF, ADF, and ADL) was conducted using the Van Soest, Robertson, and Lewis (1991) approach [28]. The method developed by Gray et al. [29] was used to determine lipids for the examination of fatty acids.

The total content of phenolic compounds was determined using the Folin–Ciocalteu reagent according to the colorimetric method described by Verma et al. [30] and Naczk and Shahidi [31]. The extraction and quantification of tocopherols was evaluated according to Rachieru et al. [32].

The color in the meat samples was measured in triplicate as the CIELAB coordinates L*, a*, and b* (CIE, 1986) using a portable colorimeter (Minolta CR300, Model CR-300, Minolta Co., Ltd., Osaka, Japan), illuminant D65, calibrated to a standard white tile.

Lastly, lipid oxidation in meat samples was assessed according to the method described by Siu and Draper [33].

### 2.5. Total Phenolic Content (TPC)

Phenolic compounds were recovered as described by Verma et al. [30] with some modifications.

Feed powder (moisture 9%) was mixed with hydroalcoholic solvent (EtOH 80%) (5:1 solvent/row material ratio). The extractions were performed on a heating plate (60 °C) with constant stirring for 60 min. Later, the produced extracts were centrifuged (10,000 rpm, 10 min, 4 °C) and the supernatant was recovered, filtered through a Buchner funnel, and stored at −21 °C until further analysis.

The total content of phenolic compounds was determined using the Folin–Ciocalteu reagent according to the colorimetric method described by Verma et al. [30] and Naczk and Shahidi (2006) [31], with appropriate modifications. Briefly, 0.5 mL of a diluted extract (1:10) was mixed with 2.5 mL of the Folin–Ciocalteu reagent (10% *v*/*v*) and 2 mL of a Na_2_CO_3_ solution (7.5% *w*/*v*). The mixture was incubated for 15 min at 45 °C and left to cool to room temperature for 30 min. Absorbance readings were taken at 765 nm against a reagent blank (reaction mixture without the sample), using a double-beam ultraviolet–visible spectrophotometer (Perkin-Elmer UV-Vis λ2, Waltham, MA, USA).

Total phenolics content was calculated using a gallic acid calibration curve (2–10 mg/L; r = 0.999) and expressed as μg of gallic acid equivalents (GAEs) g^−1^ dry matter.

### 2.6. Extraction and Quantification of Tocopherols Using HPLC Analysis

Tocopherol content was evaluated according to Rachieru et al. [32], with appropriate modifications. In total, 2 g of dried and ground feed was saponified with an ethanolic solution (55% *v*/*v*) of potassium hydroxide (11% *w*/*v*) at 80 °C for 15 min. The samples were subsequently extracted with hexane. The hexane phase was recovered and concentrated using a Rotavapor. The residue obtained was dissolved in 200 μL of mobile phase.

Injection (20 μL), after filtration through cellulose syringe filters (0.45 μm), was performed using a Knauer HPLC Smartline Pump 1000 equipped with a Smartline 2600 UV detector (Knauer Wissenschaftliche Geräte GmbH, Berlin, Germany) and a C18 reversed-phase column (250 × 4.6 mm × 5 μm). The mobile phase was 100% methanol with a flow rate of 1.5 mL/min and 40 °C temperature. Tocopherols were monitored at 292 nm, and external standards such as α-tocopherol and DL-α-tocopherol acetate (Sigma-Aldrich, St. Louis, MO, USA) were used for the quantification of tocopherols. The results are expressed as μg g^−1^ of dry matter.

### 2.7. Lipid Oxidation of Meat Samples

The LD slices were covered with an oxygen-permeable film and stored in a refrigerator at 3 °C. Analyses were conducted on the fresh product at various storage intervals: at time zero (T0), and after three (T3), six (T6), and ten days (T10).

The analysis of thiobarbituric acid reactive substances (TBARSs), used as an index of lipid oxidation, was conducted on Longissimus Dorsi muscle samples, according to the method described by Siu and Draeper (1978) [33]. Five grams of sample material was homogenized with ultraturrax (2 min at 13,000 rpm) in 15 mL of 5% (*w*/*v*) trichloroacetic acid (TCA). After filtering the homogenate through filter paper (Whatman 42), a 4 mL aliquot was taken, to which 1 mL of thiobarbituric acid (0.02 M) was added. Subsequently, after leaving the solution to incubate at 80 °C for 90 min, waiting for the development of the colorimetric reaction, the absorbance at 532 nm was measured using a double-beam spectrophotometer (Shimadzu Corporation, Milan, Italy; model UV-1800). The data were expressed in mg of MDA/kg of meat.

### 2.8. Statistical Analysis

The performance parameters and fatty acid composition data were examined using an ANOVA model (SAS 2009, SAS Institute Inc., Cary, NC, USA), with dietary treatments used as a fixed factor. The means were compared using a Tukey test, with significance determined at *p* < 0.05.

The data on meat quality were assessed through a mixed model analysis (GLM procedure), which included the effects of the diets and storage time as fixed factors and their interactions. The least-square means (LSM) and the standard error of the least-square means (SEM) were calculated. Comparisons between the least-square means were performed using the Tukey test, and the differences were considered significant at *p* < 0.05.

## 3. Results

Samples of the olive cake were collected at the end of the drying process and tested to detect the presence of mycotoxins; the results indicate negligible levels of mycotoxin contamination (well below the maximum levels set by the European Commission: Commission Regulation (EU) 2023/915) [34] in all of the olive cake samples analyzed.

As can be seen in Table 2, the greatest quantity of alpha tocopherol was found in feed in which the olive cake was present (OC and OCEL).

As expected, oleic acid (C18:1 n-9 acid) and α-linolenic acid (C18:3 n-3) were present in higher quantities in diets containing olive cake and linseed. As shown in Table 3, the dietary treatments had no influence on the carcass weight, dressing percentage, pH, average daily gain (ADG), dry matter intake (DMI), feed conversion ratio (FCR), and final body weight.

These data suggest that the partial replacement of conventional feed with olive cake does not significantly compromise animal growth, thus proving to be a sustainable option.

**Figure 1:**
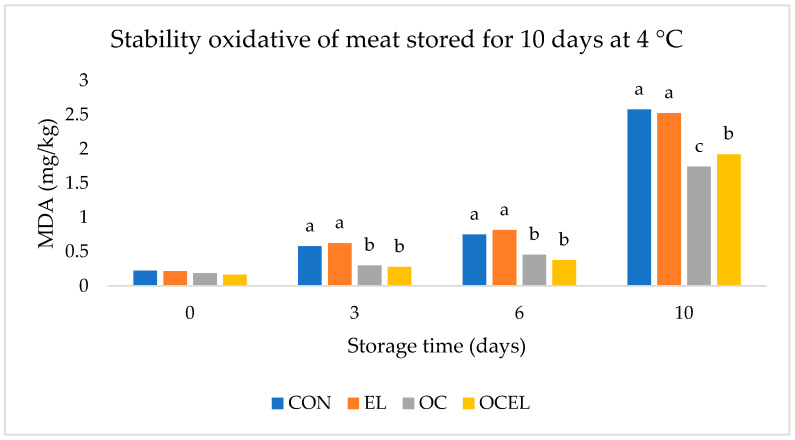
Effect of the dietary treatment and time of storage on the oxidative stability of Longissimus Dorsi. Treatments were CON = containing neither linseed nor olive cake; OC = containing 30% of stoned olive cake; EL = containing 15% of extruded whole linseed; OCEL = containing 10% of extruded whole linseed and 20% of stoned olive cake. Interactive effect of dietary treatment (CON, EL, OC and OCEL) and storage time on TBARS values measured on meat samples during aerobic storage at 4 °C. The values presented show highly significant differences (*p* < 0.001).

Regarding oxidative stability, Figure 1 illustrates the TBARSs (thiobarbituric acid reactive substances) values measured throughout the monitoring period, serving as an indicator of lipid peroxidation in the meat samples.

The control (CON) and linseed (EL) groups exhibited a marked increase in TBARS values over time, indicating a higher susceptibility to lipid oxidation. This suggests reduced oxidative stability in both groups, likely due to the absence of sufficient dietary antioxidants. In particular, despite the nutritional advantages associated with linseed, the EL group showed TBARS levels comparable to those of the control group, and was rich in alpha-linolenic acid, which may actually enhance the vulnerability of meat lipids to oxidative degradation, especially when not counterbalanced by effective antioxidant compounds.

In contrast, the OC (olive cake) and OCEL (olive cake + linseeds) groups, who were both supplemented with olive cake, displayed significantly lower TBARS values throughout the 10-day storage period. This finding reflects a pronounced antioxidative effect, which can be attributed to the natural polyphenolic compounds present in olive cake, which are known for their radical-scavenging and lipid-stabilizing properties. The ability of these compounds to delay lipid oxidation appears to compensate for the pro-oxidative nature of PUFAs when linseeds are included, as evidenced by the improved oxidative stability in the OCEL group compared with the EL group.

The color parameters monitored over the 16-day refrigerated storage period are presented in Table 4.

The dietary inclusion of olive cake (OC), linseed (EL), or their combination (OCEL) had a significant effect on the evolution of the meat color parameters during refrigerated storage, as assessed through instrumental colorimetric analysis (CIE Lab* system).

The lightness (L) of the meat was significantly influenced by dietary treatment (*p* < 0.001) and by the interaction between treatment and storage time (*p* < 0.001), whereas storage time alone had no significant effect (*p* > 0.05). At day 0 (T0), the control group (CON) showed the lowest L value, while higher values were observed in the OC, EL, and OCEL groups (*p* < 0.001). In particular, meat from the EL group displayed slightly higher L* values compared to the other groups at T0. At day 3 of refrigerated storage, the L* values remained significantly higher in the OC, EL, and OCEL groups compared to CON (*p* < 0.001). By day 6, a further decrease in lightness was observed in the CON group, while the supplemented groups maintained elevated values (*p* < 0.001). At day 10, CON still exhibited the lowest L* value, whereas the EL and OCEL groups recorded the highest lightness, followed by OC (*p* < 0.001).

The redness (a*) parameter remained consistently higher in the OC and OCEL groups compared with CON and EL across all the storage times (*p* < 0.001).

The yellowness (b*) value at day 0 was lower in CON compared with the supplemented groups (OC, EL, OCEL), with statistically significant differences (*p* < 0.001). Until time point T10, the b values remained higher in the OC and OCEL groups compared to the CON and EL groups (*p* < 0.001).

The chroma (C*), which reflects color saturation, was significantly influenced by treatment (*p* < 0.001) and its interaction with time (*p* < 0.001), while time alone did not have a significant effect (*p* > 0.05). At day 0, the CON group presented the lowest chroma value. From day 3 onwards, the chroma values in the CON and EL groups dropped below 18, whereas in OC and OCEL, they remained above this threshold through to day 10, indicating enhanced color intensity stability in the latter groups.

The hue angle (h*) increased during storage in all the groups. This parameter was significantly affected by treatment (*p* < 0.001), and its interaction with time (*p* < 0.001). At day 0, the OC and OCEL groups exhibited higher hue values than CON and EL, indicating a redder hue at baseline. At day 3, the highest values were recorded in the OCEL group, followed by slightly lower values in the OC group, and subsequently in the EL and CON groups (*p* < 0.001). At day 6, OCEL maintained the highest values, followed by the CON group, while the OC group showed values comparable to CON, and the lowest values were observed in the EL group (*p* < 0.001). At day 10, the highest values were recorded in the CON and EL groups, while the OC and OCEL groups showed slightly lower values.

## 4. Discussion

This study aimed to evaluate the effects of dietary supplementation with olive cake and linseed on the growth performance, carcass characteristics, and oxidative stability of meat in young Podolian bulls. This rustic and autochthonous breed, well adapted to marginal environments, has been scarcely explored in the context of advanced feeding strategies designed to improve both product quality and sustainability [21]. The present findings offer robust evidence supporting the inclusion of both olive cake and linseed as functional feed ingredients. These components demonstrated the capacity to enhance meat oxidative stability without impairing growth performance or carcass yield, thereby supporting their applicability in livestock systems aimed at improving resilience and energy efficiency.

Growth performance indicators—including average daily gain (ADG), final body weight, dry matter intake (DMI), and feed conversion ratio (FCR)—were not significantly influenced by dietary treatment, as demonstrated by the results in Table 3. Similarly, carcass weight and dressing percentage remained unaffected across the groups. These outcomes hold considerable relevance within sustainable livestock production models, where agro-industrial by-products are increasingly proposed as alternative feed resources to mitigate environmental impact and reduce reliance on conventional feed ingredients [35]. The absence of negative effects on animal performance confirms the suitability of both olive cake and linseed as dietary components that do not compromise either productivity or carcass quality. Importantly, the observed improvements in oxidative stability were achieved without any detriment to performance, thus meeting the dual aim of enhancing meat quality while maintaining efficiency in production.

From a qualitative perspective, this study confirmed the hypothesis that the antioxidant properties of olive cake significantly contribute to the stabilization of meat during refrigerated storage, particularly with regard to oxidative stability and color preservation. These effects are primarily attributed to the presence of bioactive compounds such as hydroxytyrosol, tyrosol, and oleuropein, which exert potent radical-scavenging and metal-chelating effects, along with naturally occurring tocopherols [36,37]. Prior research has identified the antioxidant roles of these phenolics and vitamins, which act synergistically to inhibit lipid oxidation and preserve myoglobin in its reduced forms. Supporting this mechanism, the tocopherol analysis revealed significantly elevated α-tocopherol concentrations in the groups supplemented with olive cake (OC and OCEL), as reported in Table 2, confirming the enhanced antioxidant profile of the resulting meat.

Oxidative stability and meat color are key determinants of product shelf life, marketability, and consumer preference. The visual appeal of meat, particularly its color, serves as a primary indicator of freshness, with bright cherry-red hues indicating optimal quality [38]. This attribute is closely linked to the redox state of myoglobin, which oscillates between deoxymyoglobin (purple), oxymyoglobin (bright red), and metmyoglobin (brown), as extensively described in [39]. The oxidation of oxymyoglobin to metmyoglobin results in discoloration and a corresponding reduction in commercial value [40,41,42]. Moreover, this oxidative conversion is often accelerated by the presence of reactive oxygen species (ROS), which are generated during lipid peroxidation [43].

A quantitative analysis of lipid oxidation, using thiobarbituric acid reactive substances (TBARSs), revealed that the OC and OCEL groups exhibited markedly lower values than both the control (CON) and the linseed-only (EL) groups, with highly significant differences (*p* < 0.001). These reduced TBARS levels, illustrated in Figure 1, were maintained over the 10-day refrigerated storage period, underscoring the antioxidant efficacy of olive cake. These findings align with previous studies highlighting the benefits of olive by-products in improving oxidative stability in lambs, pigs, and poultry [44], while also expanding the knowledge base regarding their effects in the underrepresented Podolian breed.

Although linseed is a well-established source of α-linolenic acid (C18:3 n-3), which enhances the n-3 polyunsaturated fatty acid (PUFA) content of meat and, thus, its nutritional profile, its high degree of unsaturation increases susceptibility to oxidation [45]. Without adequate antioxidant protection, PUFAs may accelerate lipid peroxidation, thereby compromising meat’s quality, both nutritionally and organoleptically. In agreement with this, the EL group exhibited higher TBARS levels compared with the OC and OCEL groups, reinforcing the necessity of combining PUFA sources with effective antioxidants such as those found in olive cake [46].

Colorimetric assessments supported the biochemical data. Lightness (L*) and redness (a*) values were significantly higher in the OC and OCEL groups compared to the control from day 0 and remained stable throughout storage, indicating enhanced color stability and reduced myoglobin oxidation. The EL group showed the highest L* values initially and maintained a consistently lighter meat color over time [47]. As detailed in Table 4, the observed enhancements in colorimetric parameters are particularly relevant in terms of consumer appeal, given that meat color is one of the most influential extrinsic factors affecting purchasing decisions. Specifically, cuts exhibiting higher lightness (L*) and redness (a*) values are consistently perceived as fresher and of higher quality, and are therefore preferred over darker or less vivid counterparts [48]. The sustained redness in the olive cake-supplemented groups likely reflects the protective role of dietary phenolics in preserving oxymyoglobin [49,50]. Chroma (C*), indicative of color saturation, remained above the consumer acceptability threshold of 18 until day 6 in the OC and OCEL groups, whereas this threshold was breached as early as day 3 in the CON and EL groups [51]. Hue angle (h°), which reflects the transition from red to brown hues, increased across all groups during storage, but did so more gradually in the olive cake-supplemented groups, further confirming their superior oxidative protection.

A safety evaluation of the olive cake used in this study demonstrated an absence of mycotoxins [34], validating its use as a safe and sustainable feed component. These findings are consistent with a growing body of evidence supporting the role of dietary polyphenols and natural antioxidants in improving meat stability and overall quality [52,53,54].

## 5. Conclusions

This study provides strong evidence that the dietary inclusion of stoned olive cake (OC) in the feeding regimen of young Podolian bulls effectively improves meat oxidative stability and color retention, without impairing productive performance. Indeed, the combination of stoned olive cake (OC) and linseed (EL) had no negative impact on growth performance, as key parameters, such as final body weight, average daily gain, dry matter intake, and feed conversion ratio, remained unaffected. These results confirm the feasibility of both OC and EL as functional and sustainable components in ruminant feeding strategies.

A key factor contributing to the successful outcomes observed in this study was the drying method used for the olive cake, which was carried out at ambient temperature. This gentle drying process was fundamental in preserving the bioactive compounds within the olive cake, ensuring their efficacy in improving meat quality and oxidative stability. The choice of ambient temperature drying, therefore represents an important methodological advancement for the practical use of olive cake in animal nutrition. Moreover, this system could be further optimized by adopting a greenhouse with forced ventilation powered by photovoltaic panels and incorporating floor heating through solar thermal systems, enhancing drying efficiency while maintaining sustainability and reducing energy costs.

Consequently, in diets supplemented with olive cake (OC and OCEL), the enhanced oxidative stability observed in the meat can be directly attributed to the preserved bioactive phenolic compounds, particularly hydroxytyrosol and oleuropein. These compounds effectively inhibited lipid peroxidation during meat storage and helped maintain color stability, thus enhancing meat quality traits that are essential for consumer acceptance and commercial value. In addition to its nutritional benefits, OC represents a promising model of the circular economy by valorizing olive oil industry by-products and reducing agricultural waste. However, due to the potential antinutritional effects of excessive polyphenol intake, its inclusion in ruminant diets should be carefully balanced and continuously monitored to avoid adverse effects.

Notably, the group fed exclusively with linseed (EL) exhibited higher lipid oxidation levels, underscoring the importance of antioxidant protection when using PUFA-rich ingredients. The combination of OC and linseed (OCEL) appears to be a strategic nutritional approach, offering both oxidative protection and enrichment of the meat’s fatty acid profile, in line with the growing demand for natural and health-promoting animal products.

From a sustainability perspective, the combination of olive cake and linseed constitutes a viable alternative to conventional feedstuffs, offering potential advantages in mitigating environmental impacts.

## Figures and Tables

**Table 1:** Ingredients and formulation of the experimental concentrates administered to the young Podolian bulls.

	Concentrates			
	CON	OC	EL	OCEL
Ingredient, % as-fed basis				
Corn meal	23.50	15.60	20.40	15.00
Barley meal	21.52	12.30	19.92	10.32
Wheat middlings	18.00	6.10	12.00	13.00
Broad bean (*Vicia faba minor*)	15.00	15.00	15.00	13.00
Soybean extraction meal, 46% ^3^	13.80	13.12	9.00	10.00
Extruded whole linseed	0.00	0.00	15.00	10.00
Stoned olive cake	0.00	30.00	0.00	20.00
Sugar beet pulp	4.50	4.20	5.00	5.00
Calcium carbonate	1.30	1.30	1.30	1.30
Sugar cane molasses	1.00	1.00	1.00	1.00
Lignosulfite	0.50	0.50	0.50	0.50
Sodium bicarbonate	0.40	0.40	0.40	0.40
Sodium chloride	0.10	0.10	0.10	0.10
Magnesium oxide	0.30	0.30	0.30	0.30
Mineral premix ^1^	0.05	0.05	0.05	0.05
Vitamin premix ^2^	0.03	0.03	0.03	0.03

^1^ Mineral premix contained per kilogram: Cu (copper sulfate pentahydrate), 12 mg; I (calcium iodate), 0.75 mg; Mn (manganese oxide), 45 mg; Se (sodium selenite), 0.20 mg; Zn (zinc oxide), 65 mg; Zn (zinc sulfate monohydrate), 10 mg. ^2^ Vitamin premix contained per kilogram: vitamin A (retinyl acetate), 6000 IU; vitamin D3 (cholcelciferol), 900 IU; niacin (nicotinic acid), 77.40 mg; vitamin B1 (thiamine monohydrate), 0.87 mg; vitamin B2 (riboflavin), 1.92 mg; vitamin E (DL-α-tocopherol acetate), 15 mg. ^3^ Total of 46% crude protein in the dry matter.

**Table 2:** Chemical–nutritional composition and fatty acid profile of the experimental concentrates and of the straw administered to the young Podolian bulls.

	Straw	Concentrates ^1^			
		CON	OC	EL	OCEL
Chemical composition					
Dry matter (DM), g/kg as feed	903.2	902.9	907.8	908.4	908.7
Crude protein, g/kg DM	40.3	198.5	198.5	198.5	199.0
Ether extract, g/kg DM	14.7	23.6	62.5	67.3	82.6
Ash, g/kg DM	67.4	52.7	58.2	51.9	56.8
NDF ^2^, g/kg DM	781.9	254.5	384.5	294.4	350.9
ADF ^2^, g/kg DM	501.5	75.5	207.2	94.5	158.8
ADL ^2^, g/kg DM	77.1	15.3	73.9	27.1	51.7
Net energy ^3^, MFU ^2^/kg DM	0.30	1.04	0.97	1.06	1.00
Total phenolic compounds, Tae ^4^/kg DM	1.18	6.14	15.05	7.67	14.44
Tocopherols					
Total tocopherols, µg/g DM		32.05	30.60	29.05	28.00
α-Tocopherol (%)		79.56	86.93	61.62	82.50
γ-Tocopherol (%)		18.41	11.44	37.18	16.07
δ-Tocopherol (%)		2.03	1.63	1.20	1.43
Fatty acids, % of total fatty acids					
C12:0	0.01	0.02	0.03	0.22	0.05
C14:0	2.69	1.08	1.05	1.06	1.01
C16:0	52.44	21.44	12.79	9.28	12.45
C16:1 c	0.14	0.14	0.21	0.11	0.1
C18:0	9.47	1.87	1.92	1.77	2.68
C18:1 n-9	8.44	25.96	59.87	13.82	39.57
C18:2 n-6	16.76	45.08	19.23	24.98	21.62
C18:3 n-3	4.85	1.76	1.99	45.96	19.91
C20:0	0.43	0.19	0.16	0.32	0.12
Others fatty acids	4.77	2.46	2.75	2.48	2.49
Fatty acid classes					
SFA ^2^	65.04	24.6	15.95	12.65	16.31
MUFA ^2^	8.58	26.1	60.08	13.93	39.67
PUFA ^2^	21.61	46.84	21.22	70.94	41.53
n-3 PUFA ^2^	4.85	1.76	1.99	45.96	19.91
n-6 PUFA ^2^	16.76	45.08	19.23	24.98	21.62
Fatty acid ratio					
UFA/SFA ^2^	0.46	2.97	5.10	6.71	4.98

^1^ CON = containing neither linseed nor olive cake; OC = containing 30% stoned olive cake; EL = containing 15% extruded whole linseed; OCEL = containing 10% of extruded whole linseed and 20% of stoned olive cake. ^2^ Abbreviations: NDF, neutral detergent fiber; ADF, acid detergent fiber; ADL, acid detergent lignin; MFU, meat forage units; SFAs, saturated fatty acids; UFAs, unsaturated fatty acids; MUFAs, monounsaturated fatty acids; PUFAs, polyunsaturated fatty acids; USFA/SFA, unsaturated fatty acid/saturated fatty acid ratio. ^3^ The calculations of energy values, expressed in UFC, were performed using the approach and the equations proposed by INRA (Alimentation des Ruminants; Editions Quae: Versailles, France, 2018). ^4^ Tannic acid equivalent.

**Table 3:** Performance in vita and post-slaughter measurements of young Podolian bulls receiving four different supplementations during the fattening period.

	Treatments ^1^					
	CON	OC	EL	OCEL	SEM ^2^	Significance
Items						
No. of bulls	9	9	9	9		
Start age (days)	314.50	315.63	316.75	316.03	7.66	NS
Initial weight (kg)	303.50	304.00	305.25	304.50	5.33	NS
Final weight (kg)	498.30	493.10	492.00	495.50	12.10	NS
ADG (kg/day)	1.30	1.26	1.25	1.27	0.04	NS
Hot carcass weight (kg)	264.38	260.06	260.56	263.25	10.58	NS
Cold carcass weight (kg)	259.50	255.25	255.56	258.31	10.43	NS
Hot dressing percentage (%)	52.95	52.70	52.87	53.09	0.55	NS
Cold dressing percentage (%)	51.96	51.73	51.85	52.10	0.54	NS
Cooling loss (%)	1.86	1.85	1.93	1.88	0.04	NS
Carcass conformation score ^3^	8.00 (R)	7.88 (R)	8.00 (R)	8.00 (R)	0.62 (R)	NS
Carcass fatness score ^4^	6.00	6.22	6.22	6.00	0.54	NS
pH after 1 h *post mortem*	6.71	6.66	6.67	6.62	0.08	NS
pH after 24 h *post mortem*	5.40	5.52	5.44	5.50	0.06	NS
Dry matter intake (kg/day)	8.70	8.54	8.63	8.43	0.23	NS
Feed conversion ratio (kg DM/kg gain)	6.85	7.01	7.03	6.74	0.32	NS
Feed conversion ratio (% final weight)	1.75	1.74	1.71	1.71	0.10	NS

^1^ CON = containing neither linseed nor olive cake; OC = containing 30% of stoned olive cake; EL = containing 15% of extruded whole linseed; OCEL = containing 10% of extruded whole linseed and 20% of stoned olive cake. ^2^ Standard error of the means (SEM). ^3^ According to EUROP classification, where 15 = E+ very excellent, 1 = P− very poor. ^4^ According to fatness scale, where 15 = 5+ very high, 1 = 1− very low.

**Table 4:** Variation in lightness (L*), redness (a*), yellowness (b*), chrome (C*), and hue (H*) in meat during storage 4 °C for 10 days.

Parameter	Storage Time (Days)	Dietary Treatments				SEM	Significance		
		CON ^1^	OC ^1^	EL ^1^	OCEL ^1^		F ^2^	T ^3^	F × T
L*	0	39.733 ^Ac^	41.683 ^b^	42.755 ^a^	41.819 ^Bb^	0.264	0.0001	NS	0.0001
	3	39.775 ^Ab^	42.429 ^a^	42.819 ^a^	42.537 ^Ba^				
	6	39.255 ^Ac^	41.765 ^b^	42.585 ^a^	42.678 ^Aa^				
	10	38.588 ^Bc^	42.109 ^b^	43.096 ^a^	43.194 ^Aa^				
a*	0	17.491 ^A^	18.193 ^A^	17.894 ^A^	18.189 ^A^	0.311	0.0001	0.0001	0.0001
	3	13.547 ^Bd^	16.229 ^Bb^	14.467 ^Bc^	17.289 ^Ba^				
	6	10.999 ^Cc^	14.552 ^Ca^	12.333 ^Cb^	14.967 ^Ca^				
	10	6.458 ^Db^	8.235 ^Da^	6.127 ^Db^	8.695 ^Da^				
b*	0	11.069 ^Ab^	13.405 ^Aa^	12.92 ^Aa^	13.49 ^Aa^	0.232	0.0001	0.0001	0.0001
	3	9.210 ^Bc^	12.958 ^Aa^	10.003 ^Bb^	13.040 ^Aa^				
	6	8.425 ^Cb^	11.699 ^Ba^	8.164 ^Cb^	12.202 ^Ba^				
	10	8.208 ^Cb^	9.520 ^Ca^	7.740 ^Cb^	9.741 ^Ba^				
C*	0	20.701 ^Ab^	22.601 ^Aa^	22.085 ^Aa^	22.649 ^Aa^	0.365	0.0001	0.0001	0.0001
	3	16.384 ^Bc^	20.773 ^Ba^	17.595 ^Bb^	21.656 ^Ba^				
	6	13.855 ^Cb^	18.763 ^Ca^	14.794 ^Cb^	19.313 ^Ca^				
	10	10.452 ^Db^	12.590 ^Da^	9.883 ^Db^	13.060 ^Da^				
H*	0	32.305 ^Db^	36.425 ^Da^	35.963 ^Ba^	36.594 ^Ca^	0.495	0.0001	0.0001	0.0001
	3	34.185 ^Cc^	36.629 ^Cb^	34.557 ^Cc^	37.058 ^Ca^				
	6	37.450 ^Bb^	38.777 ^Bab^	33.384 ^Cc^	39.167 ^Ba^				
	10	51.954 ^Aa^	49.165 ^Ab^	51.703 ^Aa^	48.235 ^Ab^				

Different capital letters in the same column indicate significant differences *p* < 0.0001; Different lowercase letters in the same row indicate significant differences *p* < 0.0001. ^1^ CON = containing neither linseed nor olive cake; OC = containing 30% of stoned olive cake; EL = containing 15% of extruded whole linseed; OCEL = containing 10% of extruded whole linseed and 20% of stoned olive cake. ^2^ F = Feeding. ^3^ T = Time.

## Data Availability

The original contributions presented in this study are included in the article, and further inquiries can be directed to the corresponding author.

## References

[1] Scarlet D. (2013). Annex: A New Circular Economy Action Plan for a Cleaner and More Competitive Europe. J. Chem. Inf. Model.

[2] Sherwood J. (2020). The Significance of Biomass in a Circular Economy. Bioresour. Technol..

[3] Sharma P., Gaur V.K., Sirohi R., Varjani S., Kim S.H., Wong J.W.C. (2021). Sustainable Processing of Food Waste for Production of Bio-Based Products for Circular Bioeconomy. Bioresour. Technol..

[4] Vasta V., Nudda A., Cannas A., Lanza M., Priolo A. (2008). Alternative Feed Resources and Their Effects on the Quality of Meat and Milk from Small Ruminants. Anim. Feed Sci. Technol..

[5] Gerber P.J., Uwizeye A., Schulte R.P.O., Opio C.I., de Boer I.J.M. (2014). Nutrient Use Efficiency: A Valuable Approach to Benchmark the Sustainability of Nutrient Use in Global Livestock Production?. Curr. Opin. Environ. Sustain..

[6] Salami S.A., Luciano G., O’Grady M.N., Biondi L., Newbold C.J., Kerry J.P., Priolo A. (2019). Sustainability of Feeding Plant By-Products: A Review of the Implications for Ruminant Meat Production. Anim. Feed Sci. Technol..

[7] Scerra M., Bognanno M., Foti F., Caparra P., Cilione C., De Caria P., Fortugno P., Luciano G., Natalello A., Chies L. (2023). Effect of high levels of almond hulls supplementation on performance and meat oxidative stability in lambs. Meat Sci..

[8] Scerra M., Rao R., Foti F., Caparra P., Cilione C., Natalello A., Biondi L., Bella M.S., Chies L. (2022). Influence of dietary inclusion of exhausted bergamot by-product in pigs on animal performance, fatty acid profile and oxidative stability of meat and meat products. Animals.

[9] Cifuni G.F., Claps S., Morone G., Sepe L., Caparra P., Benincasa C., Pellegrino M., Perri E. (2023). Valorization of Olive Mill Byproducts: Recovery of Biophenol Compounds and Application in Animal Feed. Plants.

[10] García-González D.L., Aparicio R. (2010). Research in Olive Oil: Challenges for the Near Future. J. Agric. Food Chem..

[11] Alcaide E.M., Nefzaoui A. (1996). Recycling of Olive Oil By-Products: Possibilities of Utilization in Animal Nutrition. Int. Biodeterior. Biodegrad..

[12] Joven M., Pintos E., Latorre M.A., Suárez-Belloch J., Guada J.A., Fondevila M. (2014). Effect of Replacing Barley by Increasing Levels of Olive Cake in the Diet of Finishing Pigs: Growth Performances, Digestibility, Carcass, Meat and Fat Quality. Anim. Feed Sci. Technol..

[13] Liotta L., Chiofalo V., Lo Presti V., Chiofalo B. (2019). In Vivo Performances, Carcass Traits, and Meat Quality of Pigs Fed Olive Cake Processing Waste. Animals.

[14] Caparra P., Chies L., Scerra M., Foti F., Bognanno M., Cilione C., De Caria P., Claps S., Cifuni G.F. (2023). Effect of Dietary Ensiled Olive Cake Supplementation on Performance and Meat Quality of Apulo-Calabrese Pigs. Animals.

[15] Estaún J., Dosil J., Al Alami A., Gimeno A., De Vega A. (2014). Effects of including olive cake in the diet on performance and rumen function of beef cattle. Anim. Prod. Sci..

[16] Caparra P., Foti F., Cilione C., Scerra M., Vottari G., Chies L. (2003). Olive cake, citrus pulp and wheat straw silage as an ingredient in lamb diets: 1. Effects on growth and carcass characteristics. Ital. J. Anim. Sci..

[17] Tzamaloukas O., Neofytou M.C., Simitzis P.G. (2021). Application of olive by-products in livestock with emphasis on small ruminants: Implications on rumen function, growth performance, milk and meat quality. Animals.

[18] Herrero-Encinas J., Blanch M., Pastor J., Mereu A., Ipharraguerre I., Menoyo D. (2020). Effects of a bioactive olive pomace extract from Olea europaea on growth performance, gut function, and intestinal microbiota in broiler chickens. Poult. Sci..

[19] Dal Bosco A., Mourvaki E., Cardinali R., Servili M., Sebastiani B., Ruggeri S., Mattioli S., Taticchi A., Esposto S., Castellini C. (2012). Effect of dietary supplementation with olive pomaces on the performance and meat quality of growing rabbits. Meat Sci..

[20] Ferrer P., Calvet S., García-Rebollar P., Blas C., Jiménez-Belenguer A.I., Hernández P., Piquer O., Cerisuelo A. (2020). Partially defatted olive cake in finishing pig diets: Implications on performance, faecal microbiota, carcass quality, slurry composition and gas emission. Animal.

[21] Molina-Alcaide E., Yáñez-Ruiz D.R. (2008). Potential use of olive by-products in ruminant feeding: A review. Anim. Feed Sci. Technol..

[22] Glasser F., Ferlay A., Chilliard Y. (2008). Oilseed lipid supplements and fatty acid composition of cow milk: A meta-analysis. J. Dairy Sci..

[23] Marino R., Della Malva A., Caroprese M., De Palo P., Santillo A., Sevi A., Albenzio M. (2019). Effects of whole linseed supplementation and treatment duration on fatty acid profile and endogenous bioactive compounds of beef muscle. Animal.

[24] Mills S., Ross R.P., Hill C., Fitzgerald G.F., Stanton C. (2011). Milk intelligence: Mining milk for bioactive substances associated with human health. Int. Dairy J..

[25] (2009). Council Regulation (EC) No 1099/2009 of 24 September 2009 on the Protection of Animals at the Time of Killing (Text with EEA relevance). Off. J. Eur. Union.

[26] (2013). European Union. Regulation (EU) No 1308/2013 of the European Parliament and of the Council of 17 December 2013 establishing a common organisation of the markets in agricultural products and repealing Council Regulations (EEC) No 922/72, (EEC) No 234/79, (EC) No 1037/2001 and (EC) No 1234/2007. Off. J. Eur. Union.

[27] AOAC (1995). Official Methods of Analysis.

[28] Van Soest P.J., Robertson J.B., Lewis B.A. (1991). Methods for dietary fiber, neutral detergent fiber, and non-starch polysaccharides in relation to animal nutrition. J. Dairy Sci..

[29] Gray I.K., Rumsby M.G., Hawke J.C. (1967). The variations in linolenic acid and galactolipid levels in Graminaceae species with age of tissue and light environment. Phytochemistry.

[30] Verma B., Hucl P., Chibbar R.N. (2008). Phenolic content and antioxidant properties of bran in 51 wheat cultivars. Cereal Chem..

[31] Naczk M., Shahidi F. (2006). Phenolics in cereals, fruits and vegetables: Occurrence, extraction and analysis. J. Pharm. Biomed. Anal..

[32] Rachieru D., Duca R., Olteanu M. (2009). Validation of a method to determine vitamin E (alpha tocopherol) from feed ingredients by HPLC using reversed phase chromatography. Univ. De Stiinte Agric. Si Med. Vet. La Lucr. stiintifice. Ser Zooteh..

[33] Siu G.M., Draper H.H. (1978). A survey of the malonaldehyde content of retail meats and fish. J. Food Sci..

[34] (2023). Commission Regulation (EU) 2023/915 of 25 April 2023 on maximum levels for certain contaminants in food and repealing Regulation (EC) No 1881/2006 (Text with EEA relevance). Off. J. Eur. Union.

[35] Chiofalo V., Liotta L., Lo Presti V., Gresta F., Di Rosa A.R., Chiofalo B. (2020). Effect of Dietary Olive Cake Supplementation on Performance, Carcass Characteristics, and Meat Quality of Beef Cattle. Animals.

[36] Martinez L., Ros G., Nieto G. (2018). Hydroxytyrosol: Health benefits and use as functional ingredient in meat. Medicines.

[37] Ponnampalam E.N., Kiani A., Santhiravel S., Holman B.W.B., Lauridsen C., Dunshea F.R. (2022). The importance of dietary antioxidants on oxidative stress, meat and milk production, and their preservative aspects in farm animals: Antioxidant action, animal health, and product quality—Invited Review. Animals.

[38] Wang Y., Domínguez R., Lorenzo J.M., Bohrer B.M. (2021). The Relationship between Lipid Content in Ground Beef Patties with Rate of Discoloration and Lipid Oxidation during Simulated Retail Display. Foods.

[39] Mancini R.A., Hunt M.C. (2005). Current research in meat color. Meat Sci..

[40] Geletu U.S., Usmael M.A., Mummed Y.Y., Ibrahim A.M. (2021). Quality of cattle meat and its compositional constituents. Vet. Med. Int..

[41] Gómez I., García-Varona C., Curiel-Fernández M., Ortega-Herás M. (2020). Effects of an extract from olive fruits on the physicochemical properties, lipid oxidation and volatile compounds of beef patties. Foods.

[42] Amaral A.B., Silva M.V., Lannes S.C.S. (2018). Lipid oxidation in meat: Mechanisms and protective factors—A review. Food Sci. Technol..

[43] Shahidi F., Zhong Y. (2015). Measurement of antioxidant activity. J. Funct. Foods.

[44] Vasta V., Luciano G. (2011). The effects of dietary consumption of plant secondary compounds on small ruminants’ product quality. Small Rumin. Res..

[45] Kaić A., Škorput D., Luković Z., Salajpal K., Kljak K., Radovčić N.M., Karolyi D. (2025). Effect of Linseed Feeding on Carcass and Meat Quality and Intramuscular Fatty Acid Profile of Simmental Bulls Slaughtered at Different Ages. Foods.

[46] Mele M., Serra A., Pauselli M., Luciano G., Lanza M., Pennisi P., Conte G., Taticchi A., Esposto S., Morbidini L. (2014). The Use of Stoned Olive Cake and Rolled Linseed in the Diet of Intensively Reared Lambs: Effect on the Intramuscular Fatty-Acid Composition. Animals.

[47] Conte G., Serra A., Casarosa L., Ciucci F., Cappucci A., Bulleri E., Corrales-Retana L., Buccioni A., Mele M. (2019). Effect of linseed supplementation on total longissimus muscle lipid composition and shelf-life of beef from young Maremmana bulls. Front. Vet. Sci..

[48] Holman B.W.B., Mao Y., Coombs C.E.O., van de Ven R.J., Hopkins D.L. (2016). Relationship between colorimetric (instrumental) evaluation and consumer-defined beef colour acceptability. Meat Sci..

[49] Domínguez R., Pateiro M., Gagaoua M., Barba F.J., Zhang W., Lorenzo J.M. (2019). A comprehensive review on lipid oxidation in meat and meat products. Antioxidants.

[50] Mokhtar S.M., Youssef K.M., Morsy N.E. (2014). The effects of natural antioxidants on colour, lipid stability and sensory evaluation of fresh beef patties stored at 4 °C. J. Agroaliment. Process. Technol..

[51] Ripoll G., Casasús I., Albertí P., Blanco M. (2011). Characteristics and meat quality evolution of veal produced in Mediterranean mountain areas. XIV Symposium of Animal Production.

[52] Muíño I., Díaz M.T., Apeleo E., Perez-Santaescolastica C., Rivas-Cañedo A., Pérez C., Cañeque V., Lauzurica S., de la Fuente J. (2017). Valorisation of an extract from olive oil waste as a natural antioxidant for reducing meat waste resulting from oxidative processes. J. Clean. Prod..

[53] Branciari R., Ranucci D., Miraglia D., Urbani S., Esposto S., Servili M. (2015). Effect of dietary treatment with olive oil by-product (olive cake) on physicochemical, sensory and microbial characteristics of beef during storage. Ital. J. Food Saf..

[54] Barriuso B., Astiasarán I., Ansorena D. (2013). A review of analytical methods measuring lipid oxidation status in foods: A challenging task. Eur. Food Res. Technol..

